# The Winding Road to Dyspnea: A Case Report of an Unusual Presentation of Anomalous Left Coronary Artery from the Pulmonary Artery

**DOI:** 10.3390/reports7040114

**Published:** 2024-12-12

**Authors:** Allen Fooks, Ranvir Bhatia, Sanjay Sivalokanathan, Neel P Chokshi

**Affiliations:** 1 Hackensack Meridian School of Medicine, Nutley, NJ 07110, USA; 2 Sports Cardiology and Fitness Program, Division of Cardiovascular Medicine, University of Pennsylvania, Philadelphia, PA 19104, USA

**Keywords:** anomalous left coronary artery from the pulmonary artery, coronary computed tomography angiography, sports cardiology, sudden cardiac death, case report

## Abstract

**Background and Clinical Significance:** Anomalous left coronary artery from the pulmonary artery (ALCAPA) is a rare congenital anomaly. Such patients are unlikely to survive adulthood without a major surgical correction. **Case Presentation:** We report a 30-year-old female with a lifelong murmur who presented to the sports cardiology clinic with progressively reduced exercise tolerance. She was eventually diagnosed with ALCAPA and underwent successful Takeuchi repair. **Conclusions:** Surgical correction is strongly recommended upon diagnosis to mitigate the associated risks and improve the prognosis for affected individuals.

## 1. Background and Clinical Significance

Anomalous left coronary artery from the pulmonary artery (ALCAPA), also known as Bland-White-Garland syndrome, is a rare cardiac defect that occurs in approximately 1 in 300,000 live births and comprises 0.24–0.46% of cases of congenital heart disease [1,2,3]. Without prompt surgical correction, 90% of infants will succumb to the disorder during their first year of life [1]. Those who survive into adulthood develop resilient collateral circulation from the right coronary artery (RCA), resulting in retrograde perfusion. However, the phenomenon of coronary steal may transpire, whereby blood is diverted from the RCA to the low-pressure pulmonary circulation, inducing subendocardial ischemia [3]. Here, we present a case of ALCAPA in a 30-year-old patient who had a lifelong murmur and presented with progressively worsening exercise tolerance. After diagnosis, the patient underwent a successful Takeuchi repair.

## 2. Case Presentation

A 30-year-old female patient presented to the sports cardiology clinic for the evaluation of chronic exertional chest pain and dyspnea, limited to one mile. She has been suffering from poor exercise tolerance since childhood and often avoided sporting activities due to the exacerbation of symptoms. At the age of 20, she was diagnosed with an unspecified murmur, but she reported a normal transthoracic echocardiogram (TTE).

She was born in Sierra Leone and migrated to the United States during her teenage years. Her past medical history includes sickle cell trait, iron deficiency anemia, latent tuberculosis (treated with 4 months of Rifampin), and pre-diabetes. Other than the contraceptive pill, she does not take any other medications. Apart from a family history of ischemic heart disease, there was no history of congenital cardiac disease or sudden cardiac death. She is a non-smoker and does not drink any alcohol or use any recreational drugs. Her baseline characteristics included a weight of 67.4 kg and a height of 1.7 m, which resulted in a calculated body mass index of 23.27. Her vitals included a heart rate of 88 beats per minute and a blood pressure of 111/71. On examination, there was a 3/6 continuous murmur, which peaked during systole. There were no features of heart failure, and her pulses were equal bilaterally in both her upper and lower extremities.

**Figure 1:**
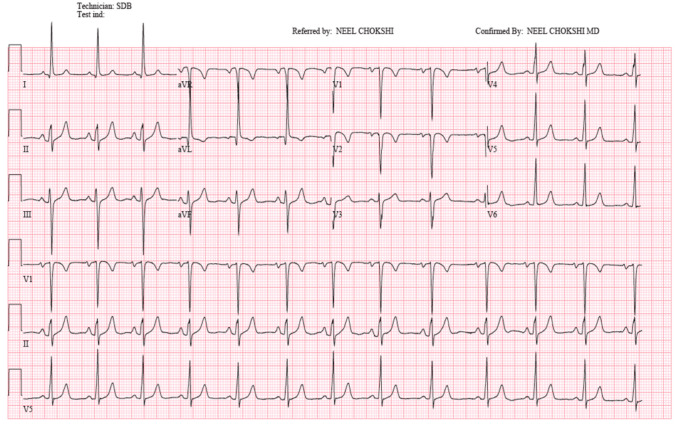
Electrocardiogram showing borderline left atrial enlargement, Q waves in V1–V2, and T wave inversions in aVL and V1–V2.

The patient’s presentation, coupled with her ECG findings (Figure 1), raised concern for myocardial ischemia. Thus, potential causes of her symptoms included coronary artery disease (CAD), coronary vasospasm, or spontaneous coronary artery dissection. However, the onset of her symptoms and the presence of a murmur suggested a congenital lesion or valvular disorder.

### 2.1. Investigations

**Figure 2:**
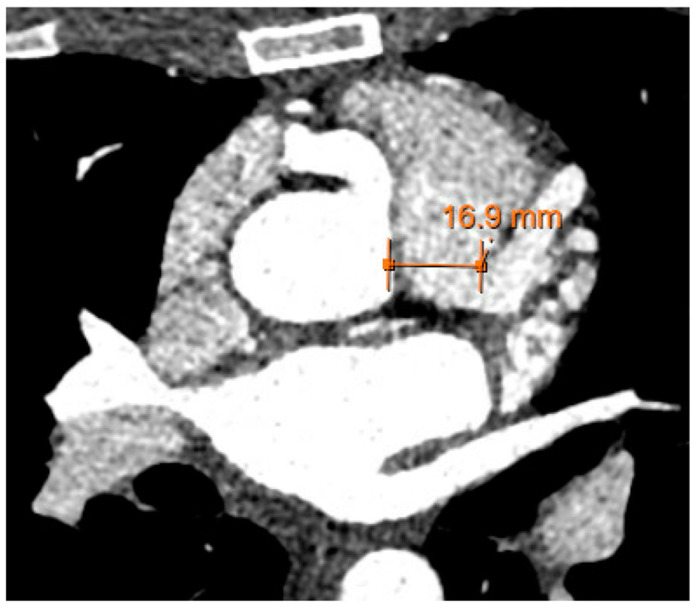
Coronary CT angiogram demonstrating anomalous origin of the left main coronary artery arising from the main pulmonary artery with a dilated right coronary artery.

**Figure 3:**
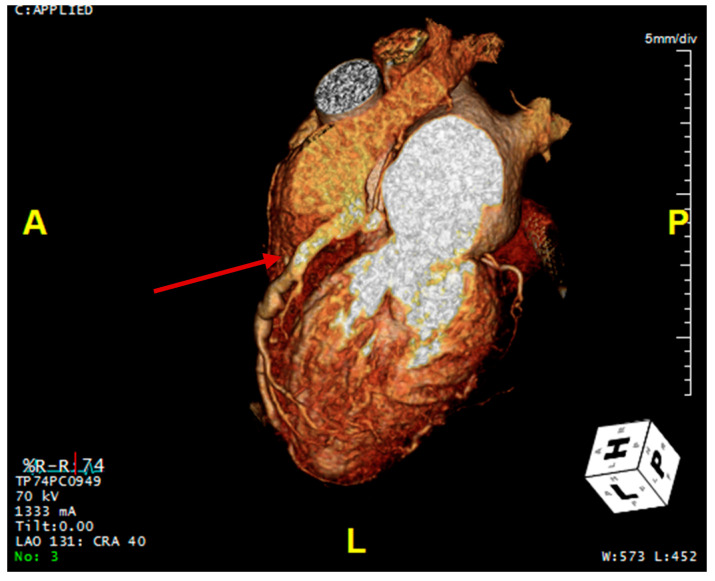
Coronary CT angiogram demonstrating the dilated right coronary artery (the right coronary artery is marked by arrow).

Transthoracic echocardiogram (TTE) revealed a normal ejection fraction, a mildly dilated left atrium, and no regional wall motion abnormalities. Here, there was color Doppler flow signal in the interventricular septum in both the 4 chamber and short axis view, suggestive of a restrictive muscular ventricular septal defect (Video S1). However, the presence of diastolic flow in the septum suggested against a ventricular septal defect. On further review, there was a prominent diastolic flow originating from the right coronary cusp with spectral Doppler consistent with coronary flow and possibly representing a coronary artery fistula of the RCA into the RV (Figure 2). More importantly, there was aneurysmal dilatation of the proximal RCA (1.0 cm), which had a highly tortuous course along the right atrioventricular groove into the posterior interventricular groove. Stress TTE with cardiopulmonary exercise testing (CPET) was performed, which revealed mild apical hypokinesis with exercise (Video S2). On exercise, the patient was able to achieve 4.2 metabolic equivalents (METs) and 66% of her predicted maximum heart rate prior to early termination due to the presence of anginal symptoms. Her peak oxygen consumption was 14.7 mL/kg/min (46% predicted). Subsequently, a computed tomography coronary angiogram (CCTA) was organized, which demonstrated an anomalous origin of the left main coronary artery arising from the main pulmonary artery (PA) with extensive collateralization from the aortic arch and the RCA as well as diffuse dilatation of the coronary arterial tree (Figure 2 and Figure 3).

### 2.2. Management

Surgical correction was pursued. Intraoperative transesophageal echocardiogram (TEE) showed aneurysmal dilation of the proximal RCA, the anomalous origin of the left coronary artery (LCA) from the root of the PA, and a Qp:Qs ratio of 2.6. Given the significant leftward insertion of the LCA on CCTA, Takeuchi repair was pursued over direct LCA re-implantation into the aorta. An aorto-pulmonary window was created prior to constructing an intra-pulmonary tunnel using a 6 mm GORE-TEX (Gore Medical Inc., Newark, DE, USA) vascular graft to baffle the aorta to the ostium of the anomalous LCA. Postoperatively, the patient was placed on apixaban for 6 months. Since surgery, she reports improved exercise tolerance and regularly follows up with cardiology for continued monitoring. 

## 3. Discussion

Reduced exercise tolerance, exertional chest pain, and dyspnea are uncommon symptoms in young adults. While atherosclerotic CAD remains the most common cause of myocardial ischemia in patients aged 15–45 years old, (responsible for nearly 90% of acute myocardial infarction within this age group), ischemia with non-obstructive coronary arteries (INOCA) is of primary concern in young adults presenting with anginal symptoms [4]. These etiologies include coronary vasospasm in the setting of substance use, spontaneous dissection of the coronary arteries, and anatomic coronary anomalies. In this setting, CCTA is an invaluable diagnostic tool.

ALCAPA encompasses two primary subtypes. The first subtype, accounting for 85% of cases, typically manifests in infancy, approximately 2–3 months post-birth, resulting in ischemic cardiomyopathy. The second subtype, presenting in adulthood, is attributed to collateralization, characterized by dilated coronary arteries [5]. Common symptoms include chest pain, dyspnea, palpitations, and syncope [6]. Upon evaluation, our patient was diagnosed with adult-type ALCAPA, featuring robust collateralization from the aortic root and RCA. The enduring continuous murmur observed was indicative of substantial left-to-right shunting across the anomalous vessel identified via TEE. Fortuitously, the collateralization facilitated the persistence of her presentation into adulthood without recognition.

According to the 2018 American Heart Association (AHA) and American College of Cardiology (ACC) guidelines, surgical repair is recommended in ALCAPA regardless of age or symptoms due to the elevated risk of ventricular arrhythmia, sudden cardiac death, and ischemia [7]. Direct reimplantation of the LCA into the aorta is the preferred surgical approach in the pediatric population, provides near-physiologic circulation, and confers the lowest rates of ostial stenosis [8]. However, it is difficult to achieve in later life due to the friability and diminished elasticity of the LCA. In this instance, the leftward insertion of the patient’s anomalous LCA made direct re-implantation a less suitable surgical strategy as there was insufficient length for a tension-free connection. Other surgical approaches commonly utilized in adults include the Takeuchi procedure, in which an intrapulmonary baffle connects the LCA to the aorta, subclavian-LCA anastomosis, and coronary artery bypass grafting with ligation of the anomalous LCA [8]. The primary complications of the Takeuchi procedure, necessitating ongoing monitoring for the patient, are baffle leaks, baffle obstruction, and supravalvular pulmonary stenosis.

## 4. Conclusions

ALCAPA remains a relatively rare congenital anomaly that typically becomes apparent at birth and can be fatal within the first year of life if not promptly addressed through surgical intervention. The condition is characterized by symptoms such as diminished exercise tolerance, arrhythmias, myocardial infarction, and heart failure. Surgical correction is strongly recommended upon diagnosis to mitigate the associated risks and improve the prognosis for affected individuals.

## Data Availability

The original contributions presented in this study are included in the article/Supplementary Material. Further inquiries can be directed to the corresponding author(s).

## Supplementary Materials

The following supporting information can be downloaded at: https://www.mdpi.com/article/10.3390/reports7040114/s1, Video S1: Transthoracic echocardiogram revealing color Doppler flow across the interventricular septum suggestive of a VSD; Video S2: Stress transthoracic echocardiogram revealing mild apical hypokinesis.
